# Misdiagnosis of otosclerosis in a patient with enlarged vestibular aqueduct syndrome: a case report

**DOI:** 10.1186/1752-1947-6-178

**Published:** 2012-07-02

**Authors:** Dayse Távora-Vieira, Stuart Miller

**Affiliations:** 1 School of Surgery, The University of Western Australia, Nedlands, Western Australia, Australia; 2 Medical Audiology Services, 51 Colin St, West Perth, Western Australia, 6005, Australia

**Keywords:** Cochlear implant, EVA syndrome

## Abstract

**Introduction:**

In the present case we report on the mismanagement of a patient misdiagnosed with otosclerosis, who was subsequently found to have enlarged vestibular aqueduct syndrome bilaterally. This highlights the need to not only be vigilant in pre-operative assessment of otosclerosis but also in post-operative investigations of stapedectomy failures.

**Case presentation:**

Our patient, a 56-year-old Caucasian Australian woman, lost the hearing in her right ear following a stapedectomy approximately 25 years ago. It is thought that preoperative imaging was not conducted, while an inadequate (unmasked) audiogram was used to formulate the initial diagnosis of otosclerosis. The hearing in her left ear deteriorated to the point that a cochlear implant was now being considered for her right ear. Imaging performed as part of our pre-cochlear implant battery revealed bilateral enlarged vestibular aqueducts and thus the decision to proceed with a right cochlear implant was made following discussion with our patient and her family in regard to not only general surgical risks but specifically the remote risk that the surgical drilling required during the procedure could risk a deterioration of the hearing in her left ear because of the enlarged vestibular aqueduct on that side.

**Conclusions:**

This report illustrates a case of misdiagnosis and mismanagement of bilateral enlarged vestibular aqueduct resulting in profound hearing loss. Fortunately our patient has been successfully implanted with a right cochlear implant with remarkable outcomes.

## Introduction

The vestibular aqueduct is a canal in the temporal bone, which extends from the vestibule to the petrous pyramid. Inside the vestibular aqueduct is the endolymphatic duct, which is connected to the endolymphatic sac. During normal embryogenesis phase the vestibular aqueduct will evolve from a diverticulum into a long and narrow canal. Enlargement of the aqueduct is associated with arrest in development of the inner ear. Hearing and vestibular symptoms have been described as clinical consequences of this anomaly, and head trauma is thought to be the most common precipitating event leading to deterioration in hearing. Valvassori and Clemis [1] were pioneers in describing the relationship between an enlarged vestibular aqueduct (EVA) and hearing loss. In their study, the incidence of EVA was 1.4%, however the resolution of imaging at that stage was unable to detect small anomalies. Boston *et al*. [2] reported that EVA explains up to 32% of the hearing loss in children who undergo temporal bone imaging.

Many adult patients with EVA may have not been diagnosed in the past due to lack of knowledge about the condition and availability of accurate imaging techniques [3]. Here, we report an interesting case of subsequent inadequate otological and audiological diagnosis leading to a profound hearing loss. Our patient has recently been successfully treated with cochlear implant.

## Case presentation

A 56-year-old Caucasian Australian woman presented to our facility with a history of unsuccessful surgery for otosclerosis in the right ear approximately 25 years ago. She says that she was told that the surgery did not work as expected, and she became deaf in that ear. At the time of surgery the surgeon reported high pressure and flow of perilymph that he characterized as a ‘gusher’. Her hearing in her left ear was already poor at the time of the operation on her right ear, and it had been deteriorating gradually. She was fitted with a hearing aid in her left ear soon after the surgery. There is no record of imaging or a previous audiogram, and it is thought that an inadequate (unmasked) audiogram was used to formulate the initial diagnosis of otosclerosis.

Our patient accepted the surgery failure and her misdiagnosis was only corrected when she decided to seek further assessment and a second opinion due to progressive deterioration of her hearing in her better ear to a level that a monoaural hearing aid was not providing enough benefit. She also reported intrusive tinnitus in her right ear and symptoms of imbalance.

A tympanometry investigation revealed normal type A tympanograms with absent ipsilateral and contralateral acoustic reflexes bilaterally. Her audiogram showed a moderate to severe sensorineural hearing loss in the left ear and a profound sensorineural hearing loss in the right ear. Word discrimination scores in quiet using AB word lists [4] were relatively poor at amplified levels. Videonystagmography was performed and caloric testing revealed a significant canal paresis in her right ear.

**Figure 1:**
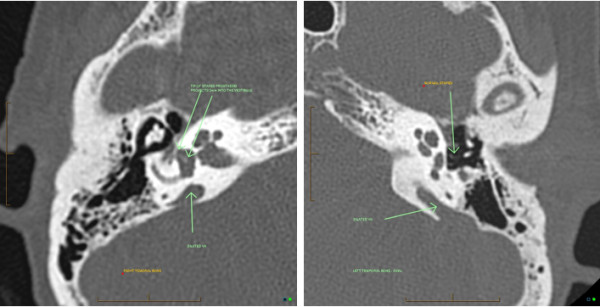
**Imaging showing bilateral enlarged vestibular aqueduct (EVA).** Normal stapes in the left ear; right stapes prosthesis is projecting 2 mm into the vestibule.

Imaging of temporal bone (Figure 1) revealed enlarged vestibular aqueduct syndrome in her right and left ears. This confirmed the previous stapedectomy in the right ear with a well positioned stapes prosthesis, and a 4 mm bony graft.

As part of our pre-cochlear implantation investigation, her hearing aid in the left ear was upgraded to improve performance. Unilateral aided outcome was assessed through speech recognition scores in quiet using the City University of New York (CUNY) sentence test [5], presented at 65dBA, 0 degree azimuth with the speaker positioned 1 m away from our patient.

The decision regarding cochlear implantation in the right ear was made with strong involvement and support from our patient’s family. They were made aware of the general risks involved in the surgery, in particular the potential for damage to her better ear from possible trauma associated with the extensive drilling during the procedure on her right ear. Our patient was implanted on 1 March 2011 using a CI24RE straight array electrode cochlear implant, and speech processor CP810 from Cochlear Ltd (Lane Cove, Australia). All electrodes were inserted, there were no short or open circuits and impedance was within the normal range. Our patient presented with a narrow dynamic range at switch on, but was able to discriminate all sounds in the Ling Sounds Test presented at soft live voice level without visual cues. Intensive auditory training was provided from day one for four weeks, using the rehabilitation material provided by Cochlear. Audiobooks with different paces of presentation were used for auditory training purposes at home. At six months follow up, open-set sentence recognition was assessed using CUNY sentences and our patient achieved a score of 96% at bimodal amplification against the initial score of 58% with hearing aid only.

## Discussion

EVA remains the most common inner ear anomaly detected in children with hearing loss who undergo imaging of temporal bone [6,7]. However, as described by Gopen *et al*. [3], there are still some controversial aspects related to this pathology including the type of hearing loss.

Sensorineural hearing impairment and mixed hearing loss with air bone gap mainly in the low frequencies are the issues most often described, and the level of deafness is variable. Hearing loss is usually progressive and, therefore, cochlear implantation may become the only option for rehabilitation. Cochlear implantation has been successfully used in children since 1995 and beneficial outcomes have been extensively reported. Among adult populations, the outcome may be advantageous due to post-lingual onset deafness.

It is thought that some complications may result from implantation in an anatomically abnormal cochlea, for instance cerebrospinal fluid leak (‘gusher’) [7,8]. Based on our patient’s case, it seems that the audiogram was inaccurate with no or poorly used masking for measurement of bone conduction thresholds and a purely conductive hearing loss was assumed. She was therefore subjected to right stapedectomy (Figure 1), which was the precipitating factor for decrease of hearing to a profound loss. In addition, it seems that imaging was not used. Hearing in her opposite ear kept deteriorating gradually, and no further investigations were undertaken as our patient was considered a hearing aid candidate soon after the surgery. Only when the hearing aid was not providing sufficient assistance, and a further opinion was sought, was the correct diagnosis of bilateral EVA made.

## Conclusions

This report illustrates a case of stapedectomy as the precipitating factor for profound hearing loss in a patient with bilateral EVA. It seems that this resulted from poor otological and audiological management. Our patient’s history suggests that lack of imaging and an adequate audiogram contributed to the initial incorrect diagnosis. This led to the unfortunate decision to proceed with middle ear surgery, which was the triggering factor for a sudden profound hearing loss. However, our patient displayed remarkable progress after cochlear implantation.

## Consent

Written informed consent was obtained from the patient for publication of this case report and any accompanying images. A copy of the written consent is available for review by the Editor-in-Chief of this journal.

## Competing interests

The authors declare that they have no competing interests.

## Authors’ contributions

DTV provided the audiological diagnosis and auditory rehabilitation. SM performed the otological diagnosis and the cochlear implantation surgery. Both authors were major contributors in writing the manuscript, and read and approved the final manuscript.
